# The Acoustic Properties of Water Submerged Lodgepole Pine (*Pinus contorta*) and Spruce (*Picea* spp.) Wood and Their Suitability for Use as Musical Instruments

**DOI:** 10.3390/ma7085688

**Published:** 2014-08-06

**Authors:** Calvin Hilde, Renata Woodward, Stavros Avramidis, Ian D. Hartley

**Affiliations:** 1 Department of Physics, University of Northern British Columbia, 3333 University Way, Prince George, BC V2N 4Z9, Canada; E-Mail: calvin.hilde@unbc.ca; 2 Ecosystem Science and Management Program, University of Northern British Columbia, 3333 University Way, Prince George, BC V2N 4Z9, Canada; E-Mail: renata.woodward@alumni.unbc.ca; 3 Department of Wood Science, the University of British Columbia, 2424 Main Mall, Vancouver, BC V6T 1Z4, Canada; E-Mail: stavros.avramidis@ubc.ca

**Keywords:** wood, acoustic constant, characteristic impedance, submerged wood

## Abstract

Wood is a common material used for the manufacture of many products, and submerged wood, in particular, has been used in niche markets and musical instruments. In order to examine if submerged wood in British Columbia, Canada, would be appropriate for use as musical instruments, a study was performed in 2007 on submerged wood from Ootsa Lake, British Columbia, Canada. The results of that study showed the wood was not suitable for musical instruments. In this paper, the wood samples were allowed to age untouched in a laboratory setting and were then retested under the hypothesis that physical acoustic characteristics would improve. It was shown, however, that acoustic properties became less adequate after being left to dry over time. This article describes the density, speed of sound, acoustic constant and characteristic impedance properties for submerged wood and a comparison is made for different applications for musical instruments.

## 1. Introduction

Wood is used in component manufacturing for musical instruments such as guitars, bagpipes, xylophones, pianos, organs and violins. The resonance characteristics of different wood species have been studied, including how to improve its acoustical characteristics, and why some wood species are more suitable than others [1]. The selection of wood has traditionally fallen upon experienced instrument makers who choose wood for maximum aesthetic and acoustical quality. Some other key wood characteristics include: absence of imperfections (*i.e.*, knots), lack of compression wood, no fungal attacks, suitable ring width and colour.

Despite the development of alternatives to wood-based products, solid wood remains the source in the manufacturing of many chordophones such as guitars, violins and pianos; aerophones, such as the oboe or bagpipes; and percussion instruments such as xylophones and drums. However, due to the non-homogeneous nature of wood, there is a large variation in properties between wood species [1] as well as between individual samples within a species that can impact the acoustic properties. Spruce, for example, is a common material in building soundboards for violins and guitars [2], due to its resonant properties [3,4,5].

Determining if wood is acoustically suitable is sometimes performed through physical means such as listening to the resonance [6]. The usual tap test is not an easy process to implement on a large-scale manufacturing of instruments. It is possible, however, to look at the physical acoustic characteristics and define what characteristics that may be required for different types of instruments.

Wood that is fully submerged in water remains in an anaerobic state and will not face attack from fungi, due to the lack of oxygen. While it is still susceptible to long-term degradation due to micro-bacterial attack, the preservation of wood from other types of degradation while under water makes submerged wood a viable supply of wood for industry. Recovered submerged wood is often used in niche markets such as for veneers for flooring [7], by local artisans for their products [7] as well as for use in the manufacturing of musical instruments [8].

In a previous experiment [2], submerged wood was tested to determine the suitability for musical instruments. The samples were obtained from Ootsa Lake, British Columbia, Canada. They were cut into circular disks and then further separated into samples with approximate dimensions of 55 mm × 15 mm × 15 mm. In that study, the wood was shown not to be suitable for musical instruments based on the acoustic constant. However, it was believed that the physical acoustic constants may improve after being allowed to age in a laboratory setting, similar to the one used for high quality musical instruments [1]. In this study, results of the acoustic measurements on the same submerged wood samples were analyzed and comparisons were made to known and accepted values of both common and resonant wood samples.

## 2. Results and Discussion

### 2.1. Speed of Sound

The speed of sound, *c_ave_*, for each disk in the 2010 data set was found to be lower compared with the 2007 data sets (Table 1). Comparing the pine and spruce samples separately showed a larger decrease in *c* of the spruce samples from 2007 to 2010 than there was for the pine samples. The difference of *c_ave_* for spruce was ~780 m·s^−1^ while the difference in *c_ave_* for pine was ~440 m·s^−1^; the differences were statistically significant. It is known that the *c* will decrease as *MC* increases [9]. For 2007 data set, the wood was *MC* = 12% and for the 2010 data set, the wood was *MC* = 6%. Therefore, the speed of sound for the 2010 data set should have been higher compared to the 2007 data set. However, the reverse was observed.

**Table 1:** Results for speed of sound measurements.

Disk—Data Set		*c_ave_* (m·s^−1^)	St. Dev.	δ*c*	Welch Two Sample *t*-test		
		–			*t-stat*	*v*	*p*-value
Pine							
1	2010	2540	207	40	−6.21	52	9.17 × 10^−08^
	2007	2910	222	40			
2	2010	2670	174	30	−9.31	56	5.82 × 10^−13^
	2007	3090	168	30			
3	2010	2680	135	30	−9.08	46	8.77 × 10^−12^
	2007	3100	200	30			
4	2010	2520	195	40	−9.49	52	6.58 × 10^−13^
	2007	3102	281	50			
5	2010	2560	198	40	−8.06	62	3.33 × 10^−11^
	2007	2990	217	40			
7	2010	2670	214	40	−4.96	52	7.99 × 10^−06^
	2007	3000	286	50			
All Pine (Average)	2010	2600	201	20	−17.97	333	<2.2 × 0^−16^
	2007	3040	245	20			
Spruce							
6	2010	2440	183	30	−12.63	49	<2.2 × 10^−16^
	2007	3230	288	50			
8	2010	2580	193	30	−15.28	64	<2.2 × 10^−16^
	2007	3340	204	40			
9	2010	2590	165	30	−18.19	59	<2.2 × 10^−16^
	2007	3500	237	40			
10	2010	2530	199	30	−13.01	67	<2.2 × 10^−16^
	2007	3230	251	30			
11	2010	2560	182	40	−12.01	47	5.70 × 10^−16^
	2007	3270	233	50			
All Spruce (Average)	2010	2540	207	40	−29.91	289	<2.2 × 10^−16^
	2007	3320	265	20			
All Samples (Average)	2010	2570	200	10	−30.87	588	<2.2 × 10^−16^
	2007	3170	291	20			

A high speed of sound is required for most resonant wood. In general, a *c* > 3000 m·s^−1^ is required while a speed of sound between 4000 and 6500 m·s^−1^ is preferred for soundboards [10]. For spruce, a *c_ave_* = 5600 m·s^−1^ (with a range 5200–6300 m·s^−1^) is chosen for musical instruments is appropriate [11] and *c_ave_* for pine is 3500 m·s^−1^[12].

In this study, both data sets were below the minimum requirements in soundboard applications. The largest measurement for the 2010 and 2007 data sets were 3050 and 3930 m·s^−1^, respectively. In addition, the *c* for the wood samples measured in 2010, were all lower than 3500 m·s^−1^, the *c_ave_* for pine [12]. This was in contrast to the 2007 data set in which both spruce and pine had values within the average range, with the average for the spruce disks being within the normal range.

### 2.2. Density

There was a statistical difference for density between the two data sets. With the exception of Disk 7, (*p*-value of 0.882), every disk showed a statistically significant difference between the two sets of measurements (95% confidence level) (Table 2). Also, between the data sets in a species group, the density values were significantly different. A decrease in density occurred for each with a difference in ρ*_ave_* for pine of ~27 kg·m^−3^ and for spruce of ~24 kg·m^−3^. Overall, the two data sets were statistically different with the 2010 data set being lower than the 2007 data, likely attributed to the lower moisture content.

**Table 2:** Results for density measurements.

Disk—Data Set		ρ (kg·m^3^)	St. Dev.	δρ	Welch Two Sample *t*-test		
		–			*t-stat*	*v*	*p*-value
Pine							
1	2010	470	32	6	−3.03	52	3.84 × 10^−03^
	2007	496	29	6			
2	2010	450	30	5	−4.45	50	4.90 × 10^−05^
	2007	480	20	4			
3	2010	508	26	5	−3.39	52	1.34 × 10^−03^
	2007	532	24	5			
4	2010	464	38	7	−4.16	58	1.05 × 10^−04^
	2007	507	39	7			
5	2010	433	41	7	−3.06	61	3.32 × 10^−03^
	2007	466	45	8			
7	2010	508	52	10	−0.15	56	0.882
	2007	510	54	10			
All Pine (Average)	2010	471	47	4	−5.46	344	9.28 × 10^−08^
	2007	498	43	3			
Spruce							
6	2010	393	22	4	−5.42	58	1.22 × 10^−06^
	2007	425	23	4			
8	2010	335	14	2	−4.13	63	1.08 × 10^−04^
	2007	351	16	2			
9	2010	429	14	2	−3.49	64	8.72 × 10^−04^
	2007	442	17	3			
10	2010	386	33	6	−3.88	70	2.33 × 10^−04^
	2007	418	36	6			
11	2010	363	31	6	−2.80	50	7.29 × 10^−03^
	2007	389	33	7			
All Spruce (Average)	2010	382	40	3	−5.12	315	5.36 × 10^−07^
	2007	406	42	3			
All Samples (Average)	2010	429	62	3	−5.17	664	3.16 × 10^−07^
	2007	454	63	3			

The desired density required for different musical instruments range between 300 and 1400 kg·m^−3^. For soundboards, a lower density is preferred, between 320 and 530 kg·m^−3^[10] whereas a narrower range has been identified for spruce between 440 and 480 kg·m^−3^[11]. Standing lodgepole pine has a density between 400 and 450 kg·m^−3^, while spruce have density between 266 and 518 kg·m^−3^ (Engelmann spruce) and 257 and 540 kg·m^−3^ (white spruce) [13]. In this study, the ρ*_ave_* values for both the 2007 and 2010 data sets were within the range of values preferred for resonant wood. The pine samples had a higher density and maximum range than the spruce samples for both data sets; both data sets had values for pine that were above normal range of resonant wood. The densities of the spruce samples, however, were below 530 kg·m^−3^[10] and 480 kg·m^−3^[11] for the 2010 data set. The spruce samples in the 2007 data set were within normal ranges for resonant wood, although the largest measurement was 486 kg·m^−3^ which was higher than the maximum 480 kg·m^−3^[11].

### 2.3. Acoustic Constant

There was a statistically significant difference between the two data sets for acoustic constant, *AC*, for each disk, for each species, and all the samples combined (Table 3). This was expected because there was a significant difference for *c* and for all, but one for ρ. There was a larger decrease in *AC* for the spruce samples compared to the pine samples. The spruce samples changed from 8.27 ± 0.09 to 6.72 ± 0.07 m^4^·kg^−1^·s^−1^ (a decrease of 1.55 m^4^·kg^−1^·s^−1^) whereas the pine *AC* changed from 6.13 ± 0.04 to 5.56 ± 0.04 m^4^·kg^−1^·s^−1^ (a decrease of 0.57 m^4^·kg^−1^·s^−1^). 

Higher *AC* values are preferred for musical instruments, primarily for soundboards that require a high speed of sound and a low density, leading to preferred values between 9 and 16 m^4^·kg^−1^·s^−1^[10]. Wood for other instruments, such as xylophone bars or violin bows, have *AC* between 4 and 8 m^4^·kg^−1^·s^−1^[10]. Recently, *AC* values of 11.15 and 10.67 m^4^·kg^−1^·s^−1^ were determined for Interior spruce from British Columbia [3].

The *AC_ave_* values for pine, spruce, and for all the samples combined were below 9 m^4^·kg^−1^·s^−1^, including error, indicating that none of the disks would be suitable for soundboards. Additionally, the *AC_max_* was 9.53 m^4^·kg^−1^·s^−1^ which was slightly above the minimum acceptable *AC*. The 2007 data set had values within the range of resonant wood (Table 3); the *AC_max_* for spruce was 9.53 m^4^·kg^−1^·s^−1^ and Disk 8 falls mostly within the range of resonant woods. Although the *AC* obtained were not appropriate for soundboards, the wood may be appropriate xylophones, violin bows, and other types of instruments meeting the minimum value of 4 m^4^·kg^−1^·s^−1^[10].

**Table 3:** Results for acoustic constant measurements.

Disk—Data Set		*AC_ave_* (m^4^·kg^−1^·s^−1^)	St. Dev.	δ*AC*	Welch Two Sample *t*-test				
		–			*t-stat*	*v*	*p*-value
Pine							
1	2010	5.41	0.46	0.09	−3.43	51	1.22 × 10^−03^
	2007	5.89	0.53	0.10			
2	2010	5.95	0.58	0.10	−3.77	49	4.45 × 10^−04^
	2007	6.45	0.39	0.07			
3	2010	5.28	0.38	0.07	−5.11	51	4.73 × 10^−06^
	2007	5.85	0.42	0.08			
4	2010	5.44	0.42	0.08	−6.96	57	3.58 × 10^−09^
	2007	6.16	0.37	0.07			
5	2010	5.95	0.50	0.09	−4.01	61	1.70 × 10^−04^
	2007	6.44	0.45	0.08			
7	2010	5.27	0.34	0.06	−5.60	49	9.48 × 10^−07^
	2007	5.91	0.50	0.09			
All Pine (Average)	2010	5.56	0.54	0.04	−29.91	289	<2.2 × 10^−16^
	2007	6.13	0.51	0.04			
Spruce							
6	2010	6.22	0.67	0.1	−6.64	52	1.77 × 10^−08^
	2007	7.65	0.94	0.2			
8	2010	7.71	0.61	0.1	−12.38	64	<2.2 × 10^−16^
	2007	9.53	0.57	0.1			
9	2010	6.04	0.41	0.07	−14.78	57	<2.2 × 10^−16^
	2007	7.94	0.61	0.1			
10	2010	6.61	0.85	0.1	−5.48	69	6.43 × 10^−07^
	2007	7.80	0.97	0.2			
11	2010	7.09	0.56	0.1	−6.57	42	6.23 × 10^−08^
	2007	8.48	0.90	0.2			
All Spruce (Average)	2010	6.72	0.89	0.07	−17.97	333	<2.2 × 10^−16^
	2007	8.27	1.07	0.09			
All Samples (Average)	2010	6.12	0.93	0.05	−30.87	588	<2.2 × 10^−16^
	2007	7.15	1.35	0.07			

### 2.4. Characteristic Impedance

The characteristic impedance, *Z*, is an important value for musical instruments, particularly for string instruments [1,10] often requiring soundboards to have a low characteristic impedance. By contrast, percussion instruments, such as xylophones, require higher characteristic impedance so that resonance will be for a longer period of time [10]. For soundboards, a *Z* value of between 1.2 and 3.392 MPa s·m^−1^ is required and between 1.68 and 5.76 MPa s·m^−1^ for woodwind instruments and xylophones [10].

From this study, the characteristic impedance was lower in the 2010 data set compared to the 2007 data set for every disk and for both pine and spruce (Table 4). These differences were statistically significant and, more specifically, the *Z_ave_* for spruce was lower than that of pine for both sets of data. This was expected since the ρ and *c* were lower in the 2010 data set.

For the 2010 data set, the *Z_ave_* value for spruce was below the accepted value for soundboards. However, the highest value was 1.26 MPa·s·m^−1^ which is within the required range. The pine samples were within range of both soundboards and wood for use in other instruments but were lower than 1.57 MPa·s·m^−1^ as reported by others [12]. The whole 2007 data set, by contrast, had values appropriate for both soundboards and other instruments.

**Table 4:** Results for characteristic impedance calculations.

Disk—Data Set		*Z_ave_* (MPa·s·m^−1^)	St. Dev.	δ*Z*	Welch Two Sample *t*-test		
		–			*t-stat*	*v*	*p*-value
*Pine*							
1	2010	1.20	0.15	0.03	−6.05	52	1.58 × 10^−07^
	2007	1.44	0.15	0.03			
2	2010	1.20	0.11	0.02	−9.65	56	1.66 × 10^−13^
	2007	1.49	0.11	0.02			
3	2010	1.36	0.10	0.02	−8.74	46	2.44 × 10^−11^
	2007	1.65	0.14	0.03			
4	2010	1.17	0.16	0.03	−7.76	51	3.46 × 10^−10^
	2007	1.59	0.24	0.04			
5	2010	1.11	0.16	0.03	−5.94	57	1.75×10^−07^
	2007	1.40	0.22	0.04			
7	2010	1.36	0.23	0.04	−2.66	55	1.02 × 10^−02^
	2007	1.54	0.27	0.05			
All Pine (Average)	2010	1.23	0.18	0.01	−22.01	283	<2.2 × 10^−16^
	2007	1.52	0.22	0.01			
*Spruce*							
6	2010	0.96	0.07	0.01	−17.48	49	<2.2 × 10^−16^
	2007	1.37	0.11	0.02			
8	2010	0.87	0.08	0.01	−13.08	59	<2.2 × 10^−16^
	2007	1.17	0.11	0.02			
9	2010	1.11	0.08	0.01	−17.48	59	<2.2 × 10^−16^
	2007	1.55	0.12	0.02			
10	2010	0.98	0.11	0.02	−12.82	65	<2.2 × 10^−16^
	2007	1.35	0.14	0.02			
11	2010	0.93	0.12	0.02	−9.18	49	3.09 × 10^−12^
	2007	1.27	0.14	0.03			
All Spruce (Average)	2010	0.97	0.12	0.01	−13.27	388	<2.2 × 10^−16^
	2007	1.35	0.18	0.01			
All samples (Average)	2010	1.11	0.20	0.01	−20.20	662	<2.2 × 10^−16^
	2007	1.44	0.22	0.01			

### 2.5. Compared Properties

**Figure 1:**
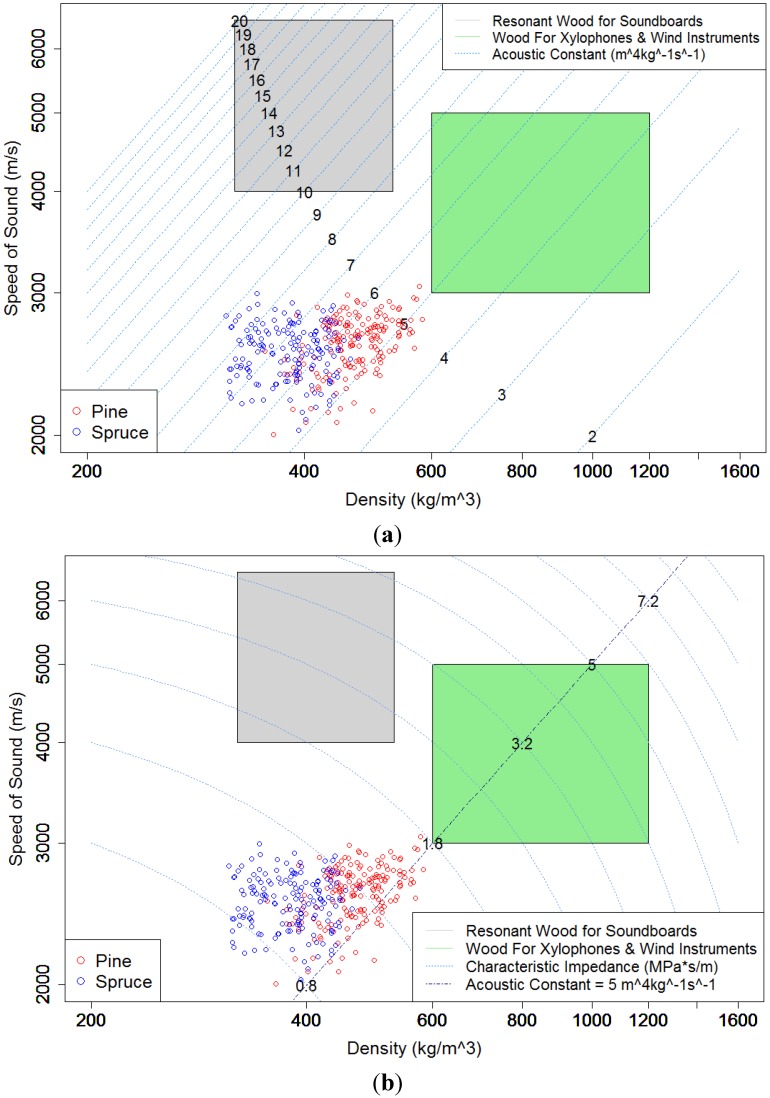
Speed *vs.* density scatterplot with acoustic constant (**a**) and with impedance (**b**). Note: logarithmic scales.

By plotting the density against the speed of sound on a logarithmic scale, known as a material property chart [10], it was possible to show how the submerged wood compared to appropriate *AC* and *Z* values for resonant woods. The accepted values for soundboards are represented by the grey and green rectangles in Figure 1a. The 2010 data set is in the accepted area. The submerged wood had a range of densities that was comparable to that of soundboards, but had noticeably lower *c* values. Based on this comparison (Figure 1a), the submerged wood did not have suitable speed of sound values and, consequently, did not have suitable *AC* values compared to soundboards. The slope of the *AC* line in Figure 1b showed the pine and spruce samples were suitable for other musical instruments [1]. Despite having an appropriate *AC* for other instruments, the *Z* value was not found to be within range of appropriate values for other instruments (Figure 1b). The submerged pine samples, however, were found to be within the range of appropriate *Z* values for soundboards even though the *AC* values were not (Figure 1a). Since the submerged pine and spruce samples have either *AC* or *Z* that are too low for soundboards or other instruments, it can be concluded that neither is appropriate for use as resonant wood.

**Figure 2:**
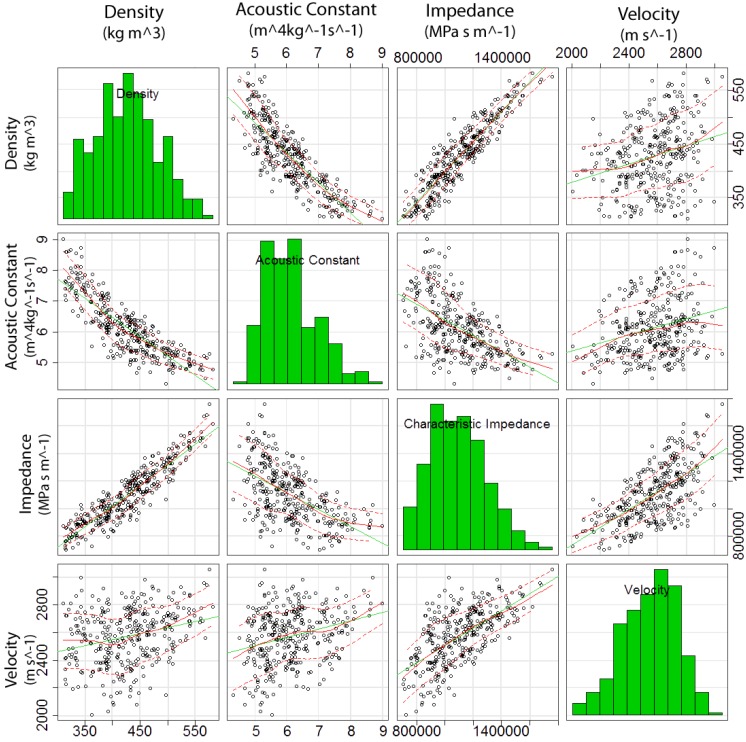
Comparison of physical acoustic characteristics.

Examining the data for ρ, *c*, *AC* and *Z* (Figure 2), the changes to *AC* were caused primarily by changes in density; that is, small changes in density lead to larger changes in *AC*. The speed, however, remained constant throughout and did not cause large variations in *AC*. This was supported by the fact that the speed of sound through wood was dependent on the density.

The dependence of *AC* on ρ can be further demonstrated by comparing changes in the *AC* and ρ for each disk individually. It was observed that the *AC* responded inversely to changes in the ρ. The same dependence was not seen when examining the acoustic constant against the speed of sound in the wood; the acoustic constant fluctuates independent of changes to the speed of sound through the wood.

The main impact that the speed of sound holds over *AC* was in its overall magnitude. When the speed of sound was high, in addition to having a low density, such as disk 10, this resulted in a much higher *AC*. The lower than average speed of sound was most likely the cause of the lower than average acoustic constant values compared to resonant woods. 

## 3. Experimental Section

Submerged trees were removed from Ootsa Lake, British Columbia, Canada (53.7500°N, 126.0333°W) in late summer 2006; the trees were submerged in fresh water in 1952 after the damming of a local river. The logs from the trees were identified as lodgepole pine (*Pinus contorta*.) and interior spruce (*Picea* spp.), which is a hybrid species of Englemann spruce (*Picea engelmannii*) and white spruce (*Picea glauca*). The average age of the trees was 133 years [2], prior to flooding.

Pine logs were numbered 1–5, 7 and 12 and spruce logs were numbered 6, and 8–11. One disk was removed from the upper part of each log and 70-mm thick disks were cut. Disks 1–5, 7 and 12 were pine and disks 6, 8–11 were spruce. From each disk, 35–40 samples were cut [2,14] with approximate dimensions of 55 mm × 15 mm × 15 mm, ensuring samples were free of defects and knots based on visual inspection. For the 2007 experimental measurements, the samples were dried in a convection oven until an equilibrium moisture content, *EMC*, of 12% was reached (determined by matched samples and by weighing to constant weight). For the 2010 experimental measurements, no additional preparation of the samples was performed except that they were stored untouched in laboratory conditions (20 °C, ~35% relative humidity) achieving an *EMC* of ~6%.

A Metriguard Stress Wave Tester (Model 239) with a pendulum steel-ball apparatus was used. Five repeated measurements were taken for each sample. The length of wood, *l* (m), that the sound wave travelled was measured and, together with average time, *t_ave_* (s), the average speed of sound, *c_ave_* (m·s^−1^) through wood was determined:

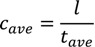
(1)

Wood density ρ*_MC_* (kg·m^−3^) is an important material property related to acoustic characteristics [1], and is calculated by:

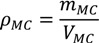
(2)
where *m_MC_* is mass (kg) and *V_MC_* is volume (m^3^) at a given moisture content, *MC*(%).

Two acoustic measurements, the characteristic impedance and acoustic constant, were determined by the combination of sound speed and wood density. The acoustic constant, *AC* (m^4^·kg^−1^·s^−1^), is a measure of the vibration within the wood as it is damped by radiating sound [1] determined by:

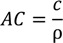
(3)

Wood impedance *Z* (Pa·s·m^−1^), is a vibration-propagation measurement between media such as from the soundboard of an instrument to the resonator [1] and is determined by:

*Z = 𝑐* · ρ (4)

The average, standard deviation and error in the average were determined using:

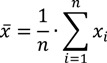
(5)
where *x* is the arithmetic mean, *x_i_* is the *i^th^* measurement and *n* is the number of measurements taken;

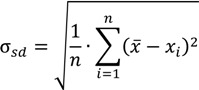
(6)
is the standard deviation; and the error of the mean is found with:

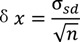
(7)

To statistically compare sets, Welch’s two sample t-statistic test was chosen due to the unequal variance between some sets and the sets being unpaired. The t-statistic (*t-stat*) was calculated using:

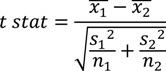
(8)
where *x*_1_ and *x*_2_ are the sample means; *s*_1_^2^ and *s*_2_^2^ are the sample variances; and *n*_1_ and *n*_2_ are the sample sizes for the first and second samples, respectively. The degrees of freedom, *v*, for Welch’s two sample t-statistic test is found using:

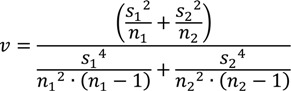
(9)

## 4. Conclusions

After an initial study performed on submerged wood revealed that the wood was not suitable for use as musical instruments, the wood was left untouched in a laboratory environment. It was believed that the physical acoustic properties of the wood, such as the density, speed of sound and, consequently the acoustic constant and characteristic impedance, would improve with aging and drying. In this study, the acoustic properties of the submerged wood, however, did not improve and instead became less desirable.

The characteristic impedance and acoustic constant of the submerged wood was compared with that of normal values for resonant wood used in soundboards, xylophones and wind instruments. It was found that a few spruce samples were within the minimum range required for the acoustic constant of soundboards but this was not the case for the characteristic impedance. Pine samples, however, had an appropriate characteristic impedance for soundboards but did not have an appropriate acoustic constant.

Both pine and spruce samples were within the acceptable range of values for acoustic constants for use in xylophones and woodwind instruments but did not have appropriate characteristic impedance. While the speed of sound was adequate for these types of instruments, both xylophones and woodwind instruments require a higher density which the submerged wood samples did not have.
